# Association between multiple genetic polymorphisms and molar-incisor hypomineralization: a population-based study

**DOI:** 10.1590/1678-7757-2025-0074

**Published:** 2025-07-21

**Authors:** Luíse GOMES-SOUZA, Aluhê Lopes FATTURI, Rafaela SCARIOT, Cleber MACHADO-SOUZA, Erika Calvano KÜCHLER, João Armando BRANCHER, Juliana FELTRIN-SOUZA

**Affiliations:** 1 Universidade Federal do Paraná Departamento de Estomatologia Curitiba PR Brasil Universidade Federal do Paraná, Departamento de Estomatologia, Curitiba, PR, Brasil.; 2 Colégio Pequeno Principe Pós-Graduação em Biotecnologia Aplicada à Saúde da Criança e do Adolescente Curitiba PR Brasil Colégio Pequeno Principe, Pós-Graduação em Biotecnologia Aplicada à Saúde da Criança e do Adolescente, Curitiba, PR, Brasil.; 3 University Hospital Bonn Medical Faculty Department of Orthodontics Bonn Germany University Hospital Bonn, Medical Faculty, Department of Orthodontics, Bonn, Germany; 4 Universidade Positivo Escola de Ciências da Saúde Curitiba PR Brasil Universidade Positivo, Escola de Ciências da Saúde, Curitiba, PR, Brasil.; 5 Universidade Federal do Paraná Programa de Pós-graduação em Biologia Molecular e Celular Curitiba PR Brasil Universidade Federal do Paraná, Programa de Pós-graduação em Biologia Molecular e Celular, Curitiba, PR, Brasil.

**Keywords:** Dental enamel, Molar hypomineralization, Genetic polymorphism, Estrogen receptor, Vitamin D Receptor

## Abstract

**Background:**

Certain genes present variants associated with molar-incisor hypomineralization (MIH) pathogenesis, especially genes encoding enamel development proteins related to morphogenesis, immune response, and hormone transcription and reception, demonstrating that MIH is likely a gene-environment issue with multiple genes having small individual effects.

**Objective:**

To evaluate the association between single nucleotide polymorphisms (SNPs) and MIH.

**Methodology:**

A sample of 90 children with MIH and 262 children without MIH were included in this study. Calibrated examiners diagnosed MIH (Kappa≥0.75) using the European Academy of Paediatric Dentistry (EAPD) criteria and modified DDE index in clinical exams. SNPs in the *IL-6* (rs2069840 and rs2069833), *ESR* (rs9340799, rs1256049, rs4986938, and rs2234693), *VDR* (rs739837 and rs2228570), and *5-HTT* genes (rs1042173 and rs38133034) were genotyped by real-time polymerase chain reaction from oral mucosa cells collected. Associations between MIH and SNPs genotypes (recessive and dominant models) and allele frequencies were tested using the chi-square test. Odds ratio (OR) and confidence intervals (CI) were calculated. A significance level of 5% was adopted. Genotypes were tested by the Hardy–Weinberg Equilibrium using chi-square.

**Results:**

In rs4986938 (*ESR2* gene), children with CT/TT presented significantly lower odds of MIH than CC (OR=0.57, CI 95% [0.35-0.92]). There was no significant association between MIH and other evaluated genes.

**Conclusion:**

The genetic polymorphism in the *ESR* gene is associated with MIH, suggesting that MIH etiology presents a polygenetic involvement.

## Introduction

Molar-incisor hypomineralization (MIH) is a qualitative developmental defect of enamel resulting from disturbances that occur during the maturation phase of amelogenesis.^1,2^ It affects the first permanent molars and may often involve the incisors, premolars, second molars, and cusp tips of canines.^1,2,3,4^ Clinically, MIH is characterized by demarcated opacities that vary in color from white to brown.^5^

Hypomineralized enamels are friable and susceptible to post-eruptive breakdown, and patients usually present dentin hypersensitivity.^5,6^ Once the enamel ultrastructure and mineral content are altered, the risk of caries increases, and adhesion of restorative materials becomes more challenging.^7,8^ Based on a recent systematic review,^9^ MIH prevalence was estimated at 13.5% worldwide. In Brazil, epidemiological studies report rates ranging from 2.5% to 40.2%.^10,11^ In this context, MIH negatively impacts the oral health-related quality of life of affected individuals and their families,^12,13^ as well as burdening healthcare systems due to increased demand for dental treatments.^14^

Currently, MIH etiology is considered complex and multifactorial, involving systemic and genetic factors acting synergistically in enamel hypomineralization.^15-22^It is well established that MIH occurs due to disturbances during mineralization or maturation stages of amelogenesis in the first permanent molars, a process that begins during pregnancy and continues throughout the first three years of life.^17,18^However, specific pathogenic pathways remain unclear, and no definitive systemic cause has been identified. Nevertheless, certain conditions during this critical period—such as maternal illness, psychological stress, cesarean delivery, birth complications, and early childhood illnesses—may be involved with MIH development.^19^ In fact, MIH exhibits characteristics of conditions with multifactorial inheritance^22^, which stems from the contribution of multiple genes and modulation by environmental factors. This is particularly evident in the asymmetrical occurrence and severity of MIH in homologous teeth. Additionally, the literature has reported a possible association between MIH and genetic variants, inherited conditions, and individual predispositions.^15,16^ Supporting this, studies have shown a higher concordance rate of MIH in monozygotic twins compared to dizygotic twins.^23,24^

Several studies^8,17,21-27^have recently observed that certain genes present variants associated with MIH pathogenesis, especially those encoding enamel development proteins related to morphogenesis, immune response, and hormone transcription and reception, demonstrating that MIH is likely a gene-environment issue with multiple genes having small individual effects.^15,22,28^ Despite efforts to investigate genetic contributions to MIH, identifying genes associated with this condition is still hampered by miscellaneous single nucleotide polymorphisms (SNPs) present in each gene. SNPs can increase the risk of a given condition or be harmless. Therefore, the literature suggests developing studies focused on understanding the mechanisms—considering genotypes and environmental risk factors combined^21,22^—to address genes and polymorphisms associated with MIH, including unusual genes.

In this context, this case-control study aimed to investigate the association between SNPs in five candidate genes with MIH: Interleukin-6 (*IL-6*)—an immune response-related gene hypothesized to interact with amelogenesis-related genes to increase MIH susceptibility;^21^ Estrogen Receptor alpha and beta (*ESR1 and ESR2*)—which have been previously studied regarding developmental enamel defects;^29-31^ Vitamin D Receptor (*VDR*)—which mediates vitamin D effects on dental mineralization and is critical in enamel formation;^32^and Serotonin Transporter (*5-HTT)*—which encodes a serotonin receptor expressed in the dental mesenchyme and epithelium*.*^33^ While these genes have been related to MIH pathogenesis, current evidence remains inconclusive, warranting further investigation.

## Methodology

### Study sample

This study was approved by the local Health Sciences Research Ethics Committee (1.613.829/2016) and the Education Department of the City Council. This city has a population of 1,908,359 inhabitants and a Human Development Index (HDI) of 0.823. Children and caregivers who agreed to participate in the study signed an Informed Consent Form. This article was reported following STREGA (STrengthening the REporting of Genetic Association Studies).^34^

A random sample was selected from a previous cross-sectional study,^35^ which comprised 736 eight-year-old schoolchildren from public schools in the city of Curitiba, located in southern Brazil. Sample size determination and sampling procedures were described in Reyes, et al.^35^ *(*2019). Children who wore orthodontic appliances that impaired vision or had syndromes associated with other enamel defects were excluded.

**Figure 1:**
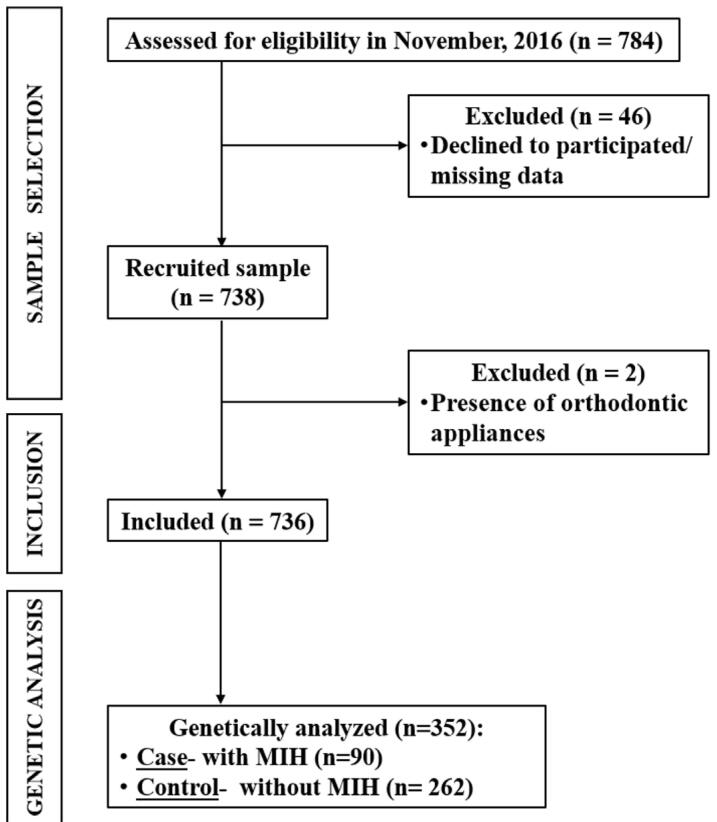
Flowchart of participants.

For this study, 352 children from the initial sample were randomly selected (Figure 1). All children with MIH were included in the case group. Children with no enamel defect were randomly selected and matched by age, ethnicity, and gender at a 1:3 ratio (case:control) to form the control group.^36^

### Clinical data collection

Clinical data was collected between November 2016 and September 2017 in a school by four trained and calibrated examiners using artificial light, a dental mirror, a dental probe, and sterile gauze. Examiner calibration is described in Reyes, et al.^35^ (2019). A modified developmental defects of enamel (DDE) index was used to diagnose MIH.^5^MIH and DDE were scored by four calibrated examiners (kappa>0.75) according to the European Academy of Paediatric Dentistry (EAPD) criteria and the FDI-modified DDE index.

### Genetic analysis

Genomic DNA was extracted from buccal cells based on a reported method^37^for molecular analysis. Genetic polymorphisms were genotyped by real-time polymerase chain reaction (PCR) using the TaqMan assay. Polymorphisms were selected based on allele frequency, linkage disequilibrium structure, and suggested association with dental fluorosis. Gene and polymorphism characteristics are described in Table 1.

**Table 1:** Characteristics of the selected genes and polymorphisms.

Gene	Gene’s role			SNP’s characteristics			
		ID	Base Change	Position	Function	Global MAF	HWE
*IL6*	Pro-inflammatory cytokines that cause airway inflammation.	rs2069840	C>G	7q11.23	Intron	0.307	0.001
		rs2069833	C>G	7q11.23	Intron	0.485	0.07
*ESR1*	Metabolism of polycyclic aromatic hydrocarbons	rs2234693	T>C	6q14.3	Intron	0.450	0.82
		rs9340799	A>G	6q14.3	Intron	0.335	0.73
*ESR2*	Influencing mineral metabolism and plasma levels of calcium, phosphorus on the metabolism of vitamin D	rs4986938	C>T	14q21.3	Missense	0.368	0.15
		rs1256049	C>T	14q21.2	Missense	0.04	0.22
*VDR*	The vitamin D receptor (VDR) mediates the effects of vitamin D on mineralization.	rs739837	G>T	12q14.1	3 prime UTR	0.487	0.06
		rs2228570	A>G	12q14.1	Missense	0.388	0.25
*5-HTT*	Development of different tissues, including tooth development and odontogenesis.	rs1042173	A>C	17q21.1	3 prime UTR	0.485	2.51
		rs3813034	A>C	17q21.1	3 prime UTR	0.449	1.72

Note: SNP = Single nucleotide polymorphism. MAF = Minor allele frequency. HWE = Hardy-Weinberg equilibrium. Retrieved from database: ncbi.nlm.nih.gov

Upon clinical examination, cells were individually collected in a private room to avoid distressing the schoolchildren. Each child performed two mouth rinses with 5 mL of autoclaved 3% glucose solution for one minute, with a five-minute interval between them.^37^ Then, oral mucosa cells were scraped with a wooden spatula to collect the patients’ DNA. Solutions were deposited in collecting tubes and stored in a polystyrene box with ice until they arrived at the molecular genetics laboratory at Universidade Federal do Paraná (UFPR), Curitiba, Paraná, Brazil. Each tube was centrifuged at 2000 rpm for 10 minutes to separate the cell pellet detached from the oral mucosa from the supernatant (saliva + 3% glucose). This cell pellet was transferred to a buffer solution (10 mM TrisHCl, 0.1 M EDTA, 0.5% SDS - pH 8.0) and stored in a freezer at -20°C until use.

Subsequently, DNA was extracted according to the Trevilatto and Line^37^(2000) protocol. After thawing, 10 µL of proteinase K (20 mg/mL) was added to the solution and incubated for eight hours at 65**°**C. The DNA was purified by adding 10 M ammonium acetate, precipitated with isopropanol and ethanol, then resuspended in 50 µl of 10 mM Tris (pH 7.8) and 1 mM EDTA. After resuspension of the DNA in Tris-EDTA or DNAse and RNAse-free water, the DNA sample was quantified using a spectrophotometer.

SNPs were selected by consulting the International HapMap Project website (www.hapmap.org)—a union aimed at developing a map with patterns of DNA sequence variations. In this database, information about the SNPs in genes of interest is available. Two SNPs (rs2069840 and rs2069833) in the *IL-6* gene, four (rs9340799, rs1256049, rs4986938, and rs2234693) in the *ESR* gene, two (rs739837 and rs228570) in the *VDR* gene, and two (rs1042173 and rs38133034) in the *5-HTT* gene were selected using the www.spedia.com website based on allele frequency (greater than 30%), and linkage disequilibrium analysis. Genetic polymorphisms were evaluated (Table 1). All SNPs selected were in Hardy–Weinberg equilibrium. The rs1256049 presents a low minor allele frequency (MAF) (0.04). Nevertheless, it was included in this study due to its relevance in various diseases, such as cancer, osteoporosis, and other metabolic conditions.^38-40^

Allelic discrimination analysis was performed for genotyping using real-time PCR and TaqMan assays (StepOnePlus Real-Time PCR System, Thermo Fisher Scientific, Waltham, MA, USA).

### Statistical analysis

The MIH dependent variable was categorized as present or absent. The presence of MIH was evaluated according to Ghanim, et al*.*^41^ (2013), when at least one molar was affected by MIH. Genotypes were categorized as dominant and recessive alleles. The chi-square test was employed to analyze the association between MIH genotypes and the genes studied, as well as to test genotypes by the Hardy–Weinberg Equilibrium (https://wpcalc.com/en/equilibrium-hardy-weinberg).

Comparisons of allele and haplotype frequencies were performed using PLINK version 1.06 (https://zzz.bwh.harvard.edu/plink/ld.shtml). Genotypic analysis was performed using Pearson’s chi-square test. Odds Ratio (OR) and 95% Confidence Interval (CI) were obtained using Poisson Regression. These analyses were performed using IBM SPSS version 25.0 (IBM Corp. Armonk, USA), and p-values <0.05 indicated statistical differences.

## Results

The sample consisted of 90 (12.21%) individuals with MIH (case group) and 262 (87.8%) without MIH (control group).

Table 2 summarizes genotype distributions among groups, considering the analysis in the genotypic and allelic models. There was no association between MIH and the SNPs studied in the *IL-6*, *ESR1*, *VDR*, and *5-HTT* genes (p>0.05). However, in the *ESR2* gene, rs4986938 was borderline in the genotypic and allele models (p=0.062 and p=0.082, respectively). Thus, rs4986938 in the recessive model was associated with MIH (p=0.020) (Table 3).

**Table 2:** Genotype and allele distribution according to the presence of MIH (Curitiba n=352).

Gene	rs#	Groups	Genotype n (%)				p-value	Allele (%)		p-value
*IL-6*			CC	CG	GG			C	G	
		2069840	Control	120(48.4)	101(40.7)	27(10.9)	0.245	341(68.8)	155(31.2)	0.697
			MIH	32(41.0)	40(51.3)	6(7.7)		104 (66.7)	52(33.3)	
				CC	CT	TT		C	T	
		2069833	Control	18(7.3)	97(39.3)	132(53.4)	0.442	133(26.9)	361(73.1)	0.277
			MIH	3(3.8)	29(36.7)	47(59.5)		35(22.2)	123(77.8)	
*ESR1*				CC	CT	TT		C	T	
		2234693	Control	45(18.1)	134(53.8)	70(28.1)	0.988	224(45.0)	274(55.0)	0.913
			MIH	12(18.2)	48(54.5)	24(27.3)		80(45.5)	96(54.5)	
				AA	AG	GG		A	G	
		9340799	Control	102(44.3)	106(46.1)	22(9.6)	0.468	310(67.4)	150(32.6)	0.944
			MIH	32(40.5)	42(53.2)	5(6.3)		106(67.1)	52(32.9)	
*ESR2*				CC	CT	TT		C	T	
		4986938	Control	97(38.2)	129(50.8)	28(11.0)	0.062	323(63.6)	185(36.4)	0.082
			MIH	47(52.2)	34(37.8)	9(10.0)		128(71.1)	52(28.9)	
				CC	CT	GG		C	T	
		1256049	Control	220(90.2)	24(9.8)	0	0.643	464(95.1)	24(4.9)	0.20
			MIH	79(91.9)	7(8.1)	0		165(95.9)	7(4.1)	
*VDR*				GG	GT	TT		G	T	
		739837	Control	58(22.8)	119(46.9)	77(30.3)	0.676	235(46.3)	273(53.7)	0.919
			MIH	18(20.9)	45(52.3)	23(26.7)		81(47.1)	91(52.9)	
				AA	AG	GG		A	G	
		2228570	Control	11(9.0)	53(43.4)	58(47.5)	0.134	75(30.7)	169(69.3)	0.202
			MIH	8(21.1)	14(36.8)	16(42.1)		30(39.5)	46(60.5)	
*5-HTT*				AA	AC	CC		A	C	
		1042173	Control	77(32.2)	95(39.7)	67(28.0)	0.528	249(52.1)	229(47.9)	0.969
			MIH	23(28.4)	38(46.9)	20(24.7)		84(51.9)	78(48.1)	
				AA	AC	CC		A	C	
		3813034	Control	36(27.5)	69(52.7)	26(19.8)	0.302	141(53.8)	121(46.2)	0.571
			MIH	18(26.5)	42(61.7)	8(11.8)		78(57.4)	58(42.6)	

Notes: PLINK compares genotypes between control and MIH groups. The frequencies between the alleles were compared using the chi-square test.

**Table 3:** Genotypic analysis of polymorphisms in dominant and recessive models according to the presence of MIH (n=352).

Gene	rs#	Model	Genotype	Groups		p-value	Odds Ratio
				MIH	Control		
*IL-6*	2069840	Dominant	CC+CG	72 (24.6)	221(75.4)	0.32	1.46 (0.58- 3.69)
			GG	6(18.2)	29 (81.8)		ref
		Recessive	CG + GG	46 (26.4)	128(73.6)	0.25	1.35 (0.80 – 1.82)
			CC	32 (21.1)	120 (78.9)		ref
	2069833	Dominant	TT+CT	76(24.9)	229(75.1)	0.271	1.99 (0.57- 6.94)
			CC	3 (3.9)	18 (85.7)		ref
		Recessive	CC+CT	32 (21.8)	115 (78.2)	0.347	0.78(0.46-1.30)
			TT	47 (26.3)	132 (73.7)		ref
*ESR1*	2234693	Dominant	CT+TT	72 (26.1)	204(73.9)	0.982	0.99(0.53-1.87)
			CC	16 (26.2)	45 (73.8)		ref
		Recessive	CC+CT	64 (26.3)	179 (73.7)	0.880	1.04 (0.60-1.80)
			TT	24 (25.5)	70 (74.5)		ref
	9340799	Dominant	AA+AG	74 (26.2)	208 (73.8)	0.380	1.57(0.57-4.28)
			GG	5 (18.5)	22 (81.5)		ref
		Recessive	AG+GG	47 (26.9)	128 (73.1)	0.552	1.17(0.69-1.97)
			AA	32 (23.9)	102 (76.1)		ref
*ESR2*	4986938	Dominant	CT+CC	81 (26.4)	226(73.6)	0.788	1.11 (0.50- 2.46)
			TT	9 (24.3)	28 (75.7)		ref
		Recessive	CT+TT	43(21.5)	157(78.5)	**0.020**	**0.57(0.35-0.92)**
			CC	47(32.6)	97(67.4)		ref
*VDR*	739837	Dominant	GT+TT	68 (25.8)	196 (74.2)	0.714	1.04(0.62- 2.04)
			GG	18 (23.7)	58 (76.3)		ref
		Recessive	GG+GT	63 (26.2)	177 (73.8)	0.530	1.19(0.69-2.06)
			TT	23 (23.0)	77 (77.0)		ref
	2228570	Dominant	AG+GG	30(21.3)	111 (78.7)	0.045	0.37(0.14-1.01)
			AA	8 (42.1)	11 (57.9)		ref
		Recessive	AG+AA	22 (25.6)	64 (74.4)	0.557	1.24(0.60- 2.63)
			GG	16 (21.6)	58 (78.4)		ref
*5-HTT*	1042173	Dominant	AA+AC	61 (26.2)	172 (73.8)	0.559	1.19(0.67-2.12)
			CC	20 (23.0)	67 (77)		ref
		Recessive	AC+CC	58 (26.4)	162 (73.6)	0.521	1.20(0.69-2.09)
			AA	23 (23.0)	77 (77.0)		ref
	3813034	Dominant	AA+AC	60 (36.4)	105 (63.6)	0.151	1.86(0.79-4.36)
			CC	8 (23.5)	26 (76.5)		ref
		Recessive	AC+CC	50 (34.5)	95 (65.5)	0.879	1.05(0.54-2.04)
			AA	18 (33.3)	36 (66.7)		ref

Notes: PLINK compares genotypes between dominant and recessive models using the chi-square test. Bold values mean p-values <0.05.

Individuals carrying CT/TT in the *ESR* gene, rs4986938, had lower odds of developing MIH than CC, suggesting a protective effect of CT/TT.

## Discussion

MIH is a complex condition with multifactorial etiology, and since biological mechanisms involved are not yet fully understood, pathogenesis pathways may be diverse.^42^ Regarding inherited conditions related to MIH, several studies have demonstrated that multiple genetic or epigenetic components may contribute to the condition. Thus, genetic variability for MIH occurrence has been widely evidenced in the literature.^19,21,22,27,43,44^ The most investigated SNPs related to MIH are from genes that encode enamel proteins, such as *AMBN*, *AMELX*, *ENAM*, *TUFT1*, *MMP-20*, *TFIP11*, *KLK4*, *ITGB6*, *LAMA3*, *LAMB 3*, *FAM83H*, *DLX3*,^17,23,26,45,46^ and *AMTN.*^46^ Moreover, the coordination and genetic control of enamel development proteins involve signaling pathways from over 300 genes^47^—all of which are potential candidates for further investigation.

Still exploring the multifactorial complexity of MIH, SNPs in less common candidate genes—such as immune-related genes (*TGFBR1*, *TGFA*,^20^ *TNFRSF11A*, and *IL10RB*^47^),^18,21,46^ endocrine genes (*VDR*^8,46,49^), and xenobiotic detoxification genes (*ARNT*, *CYP1B1*, and *ESR1*)^46^—may also influence amelogenesis, revealing an intricate genetic network in dental development. Given this context, the association between immune response SNPs and MIH was investigated.^21,46^ Bussaneli, et al.^21^ (2019), in their family-based study, evaluated whether genes related to immune response and amelogenesis were associated with MIH susceptibility. Results showed an association between the *TGFBR1* gene and severe MIH, suggesting that alterations in immune response genes may influence proper enamel development. Also, beyond SNPs in genes directly related to enamel formation, polymorphisms in immune response genes might have a synergistic effect and increase chances of developing MIH. In a study^46^ involving 659 Lebanese children, 51 genes were analyzed as risk factors for MIH. Among these studied genes, *TNFRSF11A* and *IL10RB*, involved in immune response and fever regulation, were significantly associated with MIH. In this study, *IL-6* SNPs, rs2069840, and rs2069833 showed no association with MIH. Although there are no data about the association of these specific SNPs with MIH, this finding aligns with other studies investigating different *IL-6* SNPs.^21,46^ Given the diversity of genes and SNPs underlying immune response, other immune response polymorphisms should be studied.

A systematic review published in 2024 reported that genes such as *IL-6*, *ESR*, *VDR*, and *5-HTT* are associated with MIH.^43^ In this study, we evaluated whether there was an association between SNPs and MIH in a sample of Brazilian children. *IL-6* was selected as a target gene due to its genetic susceptibility to adverse neurodevelopmental outcomes associated with preterm birth,^48^ which may be related to imbalances in mineralization or maturation of amelogenesis. Both rs2069840 and rs2069833 tested in this study are intronic SNPs that often contain regulatory elements such as enhancers, silencers, or transcription factor binding sites. This makes them natural candidates for further investigation, even though no association between these SNPs and MIH was observed. Next, we analyzed *5-HTT*, a solute carrier family gene with many MIH-associated SNPs,^49^ which is expected given its involvement with bone metabolism^50,51^ and development, growth, and formation of different tissues, including teeth.^52^Baudry, et al.^53^ (2019) discovered a serotonin concentration gradient from a vascular source of serotonin to low-affinity uptake sites in the invaginated dental epithelium. The presence of serotonin in both the dental mesenchyme and epithelium supports the hypothesis that serotonin plays a role in tooth development.^54^ Considering that *5-HTT* is not usually associated with tooth mineralization, we selected two intronic SNPs with high MAF—rs1042173 and rs3813034—but neither showed association with MIH. Therefore, these SNPs should not be prioritized in future research.

The literature shows that *ESR* genes have been extensively studied because signaling pathways mediated by estrogen hormones depend on the interaction with estrogen receptors encoded by these genes. There are two subunits of *ESR* genes: *ESR1*, which encodes estrogen receptor alpha (ERα), and *ESR2*, which encodes estrogen receptor beta (ERβ). *ESR1* is related to the detoxification of xenobiotics and has been associated with a higher MIH occurrence.^43^ *ESR1* is one of the genes responsible for the metabolism of polycyclic aromatic hydrocarbons, and it is expressed after exposure to environmental pollutants.^55^ SNPs in *ESR1* may alter the metabolism and amount of chemicals excreted, which can negatively affect cells.^46,56^ In this study, rs2234693 and rs9340799 in *ESR1* were not associated with MIH in any genetic model. Considering that the sample size is significant and the allele frequency of the rarest allele of these two SNPs is high (over 0.30), our results suggest that these SNPs are not involved in MIH.

As for *ESR2*, we selected two SNPs: rs1256049 and rs4986938. The former was previously tested for delayed tooth eruption,^57^variability in tooth crown size,^58^ and dental age,^59^ with no significant associations reported. Similarly, it was not associated with MIH in our sample. The frequency at which the second most prevalent allele appears in a population is very low (0.04), and future studies should consider testing this association in other populations. However, rs4986938 was associated with MIH. While a borderline association with MIH was observed in the genotypic and allelic models, rs4986938 protected against MIH development (p=0.020; OR=0.57 [0.35-0.92]) in the recessive model. This finding aligns with existing evidence suggesting that *ESR2* may modulate the activity of ameloblasts and odontoblasts during tooth development, highlighting the role of estrogen signaling pathways in enamel formation and mineralization.^21,35^ It further supports the biological plausibility of including *ESR2* in studies investigating qualitative developmental defects of enamel. Moreover, both analyzed SNPs are missense variants, which may change the tertiary or quaternary structure of the ESR2 receptor. Such changes could reduce estrogen binding affinity, thereby impairing signaling pathways critical for proper tooth mineralization.

Similar to *ESR1*, the *VDR* gene is involved in tooth development. It encodes the vitamin D receptor, which is expressed in odontoblasts and is crucial in regulating the expression of dentin matrix proteins. These proteins are essential for the proper formation, structure, and mineralization of dentin.^47^ The mechanism by which vitamin D stimulates tooth enamel mineralization involves binding to VDRs, which are expressed in dental and bone cells.^32^ A previous study showed an association between rs78783628 in *VDR* and MIH.^46^Fatturi, et al*.*^8^ (2020) conducted a study with children affected by MIH and found that those with the GT/GG genotype in the rs739837 *VDR* gene had a higher prevalence of MIH in molars and incisors compared to individuals with the TT genotype. However, no association was observed between MIH and rs2228570, which aligns with our findings (OR=0.37 [0.14-1.01]).

Finally, this study had strengths and limitations. Strengths include a coherent logic and standardized methodology for diagnosing MIH and a cohort based on a representative population sample. Limitations include no analysis of different MIH severities, a limited number of genes related to enamel formation, and no interaction analysis between groups of genes and environmental factors. Regarding statistical approach, we chose to report unadjusted p-values to emphasize the exploratory nature of our findings. Based on the literature,^60^we believe this approach more appropriately balances risks of Type I and Type II errors given our study design and objectives. Nevertheless, we recognize this choice may increase the likelihood of Type I errors, which should be considered a potential limitation when interpreting our results. These interactions can be evaluated in future studies, as identifying genes associated with MIH is important to elucidate mechanisms underlying hypomineralized enamel formation. Additionally, it can contribute to future studies on gene therapies aimed at preventing this enamel development defect. Thus, investigating MIH etiology remains a significant challenge, and future studies should be designed to consider genotypes and environmental risk factors combined.

## Conclusion

Despite the limitations of this study, the findings suggest that the SNP in ESR (rs4986938) is significantly associated with MIH. Children carrying the CT or TT genotypes showed a significantly lower risk of developing MIH compared to those with CC genotype.
